# Adaptive Low-Power Listening MAC Protocol Based on Transmission Rates

**DOI:** 10.1155/2014/473132

**Published:** 2014-08-28

**Authors:** Kwang-il Hwang, Gangman Yi

**Affiliations:** ^1^Department of Embedded Systems Engineering, Incheon National University, Incheon 402-772, Republic of Korea; ^2^Department of Computer Science & Engineering, Gangneung-Wonju National University, Gangwon-do 220-711, Republic of Korea

## Abstract

Even though existing low-power listening (LPL) protocols have enabled ultra-low-power operation in wireless sensor networks (WSN), they do not address trade-off between energy and delay, since they focused only on energy aspect. However, in recent years, a growing interest in various WSN applications is requiring new design factors, such as minimum delay and higher reliability, as well as energy efficiency. Therefore, in this paper we propose a novel sensor multiple access control (MAC) protocol, transmission rate based adaptive low-power listening MAC protocol (TRA-MAC), which is a kind of preamble-based LPL but is capable of controlling preamble sensing cycle adaptively to transmission rates. Through experiments, it is demonstrated that TRA-MAC enables LPL cycle (LC) and preamble transmission length to adapt dynamically to varying transmission rates, compensating trade-off between energy and response time.

## 1. Introduction

Wireless sensor networks (WSN) have been evolved drastically over the past few decades. Most research on WSN has focused on energy conservation in various aspects of hardware, protocols, schedulers, sensing, and so forth. In particular, it is well known that designing energy efficient protocol is the most critical factor for wireless sensor networks, since eventually sensory information obtained from various sensor nodes should be transmitted to a central sink or server over the network. Many researchers pointed out energy inefficiency of conventional wireless network technologies (in particular idle listening problem, etc.), and they also made attempts to devise new energy efficient protocols. Eventually these efforts to minimize energy usage in wireless communication systems have enabled ultra-low-power sensor network operation using various low-power listening (LPL) protocols. The main goal of low-power listening is to reduce unnecessary listening period of wireless receiver. According to the triggering methods, LPL protocol can be categorized into three types: preamble-based LPL [1–3], packet-based LPL [4–6], and receiver-initiated LPL [7, 8]. Preamble-based LPL utilizes long or consecutive short preamble transmission to trigger receivers that are performing periodic preamble listening. On the other hand, packet-based LPL is based on consecutive data packet or dedicated control packets to trigger receivers. Some LPL protocols utilize not a sender but a receive-initiated communication.  Table 1 summarizes the brief features and characteristics of representative LPL protocols. In particular, Hwang and Jang [9] emphasized that preamble-based LPL protocol can save much more energy than packet-based LPL protocol through experiment, and these LPL protocols are suitable for a cluster-based sensor network [10]. In addition, Yoon et al. [11] proposed a message-rate based energy efficient sensor network protocol.

Even though the existing LPL protocols have enabled ultra-low-power operation in WSN, they do not address trade-off between energy and delay, since they focused on energy efficiency itself. However, in recent years, a growing interest in various WSN applications is requiring new design factors, such as minimum delay and higher reliability, as well as energy efficiency.

Therefore, in this paper, we propose a novel sensor MAC protocol, transmission rates based adaptive low-power listening MAC protocol (TRA-MAC), which is a kind of preamble-based LPL but is capable of controlling preamble sensing cycle adaptively to transmission rates. So, TRA-MAC can cope well with concurrent-intensive traffic environment in WSN by coping well with trade-off between delay and energy.

The major contribution of the TRA-MAC is that the TRA-MAC tries to address trade-off between energy and delay, while most existing LPL protocols focused only on energy aspect. It is also important to meet various requirements, such as minimum delay and higher reliability, as well as energy efficiency. Therefore, the TRA-MAC is designed to cope well with concurrent-intensive traffic environment in WSN by dealing with trade-off between delay and energy.

## 2. Transmission Rate Based Adaptive Low-Power Listening MAC Protocol Design

### 2.1. Overview

In order to minimize unnecessary energy wastage, a preamble-based LPL node repeats preamble sensing for short active duration and sleeping for the rest of duration, within a listening cycle (LC). Preamble-based LPL can maximize energy conservation by lengthening sleep duration, but data response time might be significantly delayed, since a sender should transmit a long preamble to trigger a receiver which is performing periodic preamble sensing with long LC.

On the other hand, if too short preamble is used, data response time can be reduced but energy might be wasted. That is, there exists a trade-off between energy and delay. Therefore, the key idea of the proposed LPL protocol, transmission rate-based adaptive low-power listening MAC (TRA-MAC), is to make it possible for LC to adapt dynamically to traffic variations. That is, devices in frequent communications shorten LC to improve data response time, but devices in infrequent communications lengthen LC to conserve energy. This might result in compensating trade-off between energy and delay.

To manage communication frequency of each node and make it possible to vary *L*_preamble_ adaptively according to communication frequency, TRA-MAC employs a device management table (DMT), which contains communication frequency information of each node. TRA-MAC basically considers a star topology in which a master node (coordinator in WPAN) manages a number of slave nodes (devices), so DMT of devices is managed by a master node. That is, request-oriented data transfer model is considered. Master is capable of assessing communication frequency, managing DMT, and assigning new LC value to devices. Therefore, devices in frequent communications can perform LPL with short LC and devices in infrequent communications can perform LPL with longer LC. The rest of this section presents how to assess transmission rates, how to determine LPL cycle bound, and how the TRA-MAC works.

### 2.2. Transmission Rate Assessment

**Figure 1:**
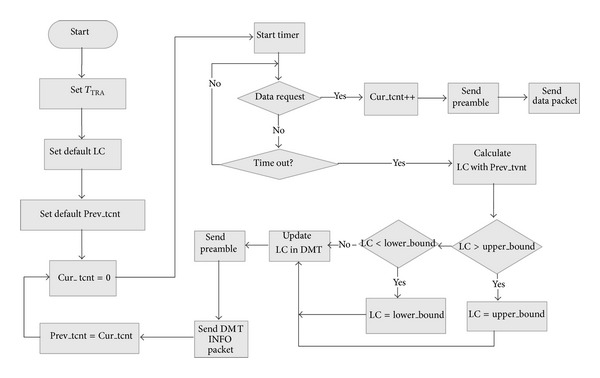
Transmission rate assessments.

Transmission rate assessment (TRA) is performed by master device which manages DMT. For continuous transmission rate assessment of each device, master counts communication frequency of each device for each *T*_TRA_.* Cur_tcnt*(*j*) is used to represent communication frequency of node *j* for the present TRA duration. At the end of each TRA, master compares* Cur_tcnt*(*j*) with* Prev_tcnt*(*j*), which represents communication frequency that occurred for the previous TRA duration. Therefore, new LC value in DMT is updated based on transmission rate and then the updated DMT result is forwarded to the devices.  Figure 1 describes more detailed TRA procedure. At initial phase, default TRA duration and LC are configured by administrator, and* Cur_tcnt* is set to “0.” At the beginning of new TRA, TRA timer starts, and whenever transmission to node *j* occurs,* Cur_tcnt*(*j*) in DMT is increased. To trigger receiver (*j*), a sender should transmit a preamble longer than LC(*j*), so *L*_preamble_ (*j*) is determined by LC(*j*) + 2∗*T*_PSD_. If a sender has no LC information of a receiver, preamble transmission of the sender lasts for maximum preamble length, and then the sender starts to count* Cur_tcnt* of the receiver to calculate new transmission rate of the receiver. Whenever timeout event of TRA timer occurs, master updates LC value of each device in DMT based on* Cur_tcnt*(*j*) and* Prev_tcnt*(*j*), for all *j* ∈ *D (set of devices managed by the mater)*. To reflect transmission rates from previous TRA until present TRA, new LC(*j*) is updated as follows:

(1) $$\begin{array}{c} \text{L}{\text{C}}_{\left(i\right)}=T_{\text{TRA}}\left(\frac{\alpha }{Cur\_tcnt_{\left(j\right)}+1}+\frac{1-\alpha }{Prev\_tcnt_{\left(j\right)}+1}\right), \end{array}$$

where *α* is weight coefficient.

In general, taking *α* greater than 0.5, new LC value becomes more sensitive to the latest communication frequency than the previous one. If master completes DMT update of all devices at every update phase, the updated LC information should be broadcasted to devices. Therefore, based on the received LC information, LC of each node is varied adaptively to the rates of communications that the node attends. That is, when communications occur frequently, the node performs periodic preamble sensing with small LC value to cope quickly well with communication requests, and when communication rarely occurs, the node performs periodic preamble sensing with longer LC to save much more energy. Like this, TRA-MAC utilizes adaptive LC of each node based on transmission rate assessment.

### 2.3. LPL Cycle Bound

TRA-MAC makes LC of each node adaptive based on transmission rate assessment. However, if communication of a node occurs continuously, its LC might be decreased more and more. On the other hand, if communication of a node does not occur for a long time, its LC might be increased more and more. It is important to note that those situations might cause serious problems as follows. In the former case, too small LC might make frequent device wake-up and therefore active duration is lasted for a long time. Eventually, it results in significant energy consumption. On the other hand, in the latter case, too long LC requires longer preamble length (*L*_preamble_) of a sender, so that data response time might be significantly delayed when a new communication request occurs.

Therefore, to avoid the infinite increment or decrement of LC, we bound the LC value as follows. Increasing LC is bounded to upper bound (UB), which depends on maximum delay acceptable in application, and obtained as follows:

(2) $$\begin{array}{c} \text{MDT}=\text{UB}. \end{array}$$

Decreasing LC is also bound to lower bound (LB), and thus unnecessary energy wastage is minimized. LB is obtained as follow:

(3) $$\begin{array}{c} \text{LB}=\text{L}{\text{C}}_{\min}=2*T_{\text{TRA}}. \end{array}$$

As shown in Figure 1, in each update phase, master checks whether newly updated LC value is greater than UB or lower than LB. So, finally, bounded LC is assigned to the device.

## 3. TRA-MAC Operation

**Figure 2:**
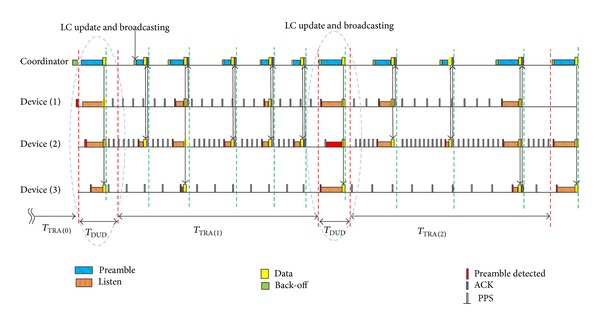
TRA-MAC operation.

Figure 2 illustrates an example operation of TRA-MAC, forming a star topology by a coordinator and 3 devices. In this example, TRA duration is set to 10 seconds and the rest of parameters are set as in Notations section.

Table 2(a) shows LC value of each device updated at the end of *T*_TRA_ (0). Based on each LC value obtained at *T*_TRA_ (0), during *T*_TRA_ (1) devices (1), (2), and (3) perform periodic preamble sensing at different LC (3.25, 2.58, and 4.83 sec), respectively, as shown in Figure 2. During *T*_TRA_ (1) device (1) has 1 communication, device (2) has 4 communications, and device (3) has 1 communication. In particular, each device is operating with different LC so that coordinator uses preamble with different length to trigger the corresponding device. Note that short preamble used to trigger device (2) is rarely affected by other devices, which are operating with longer LC. At the end of *T*_TRA_ (1), DMT is updated again as shown in Table 2(b). In particular, during *T*_TRA_ (1), device (1) communicated frequently, so actual LC value calculated by (1) is smaller than lower bound (LB), so the LC value is replaced with LB. In addition, calculated LC value of device (3), which has only two communications during *T*_TRA_ (0)* and T*_TRA_ (1), is greater than upper bound (UB), so the LC value of device (3) is replaced with upper bound (UB). Therefore, DMT of each device is represented as shown in Table 2(b), and during *T*_TRA_  (2) each device operates with the updated LC value. Like this, TRA-MAC enables each device to adapt its LC dynamically according to varying transmission rates, and in particular, the LC values below or above the thresholds are bounded within a certain range not to deteriorate network performance, compensating trade-off between energy and response time.

## 4. Performance Evaluations

**Figure 3:**
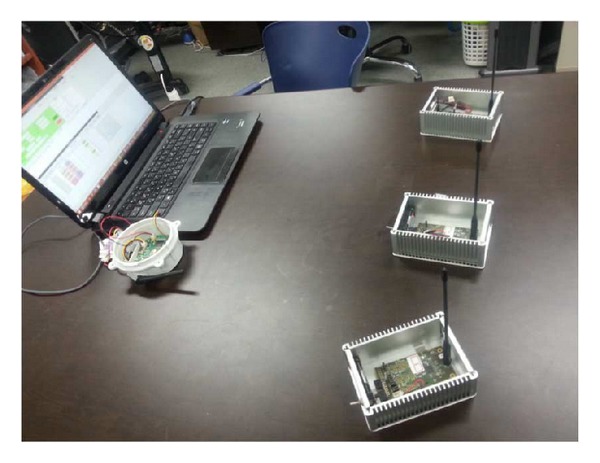
TRA-MAC test bed.

As shown in Figure 3, we developed a TRA prototype, which is based on TI CC430F6137 ultra-low-power RF SoC [12]. On the prototype hardware, hardware abstraction layer (HAL) is constructed and then LPL protocol capable of preamble transmission of variable length is implemented. A test bed composed of TRA prototypes, in which a coordinator (master) and 3 devices are used, is used to evaluate the performance of TRA-MAC compared to generic preamble-based LPL protocol (fixed-length preamble). Our evaluations include adaptivity, response delay, and energy consumption with respect to variable data traffic.

### 4.1. Adaptivity

**Figure 4:**
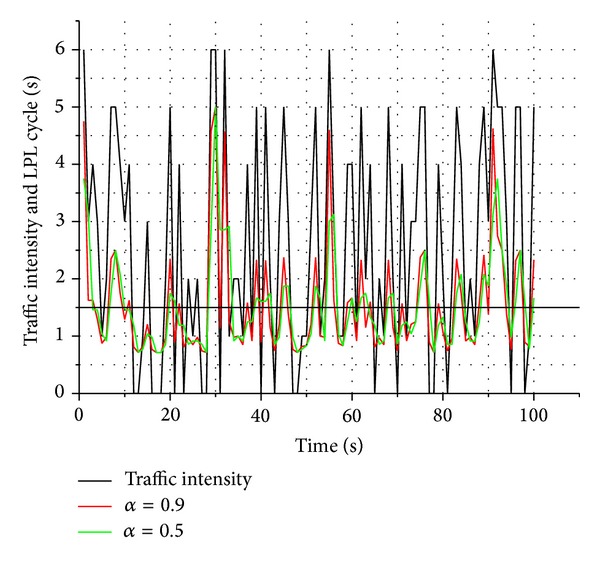
LC variation versus traffic intensity.

The main goal of TRA-MAC is to adaptively control LPL cycle with respect to transmission rates. Therefore, for adaptivity test, we observed how well the LPL cycle follows the dynamically varying traffic. In particular, as mentioned in Section 2.2, since LPL cycle depends on *α*, weight coefficient, in (1), our experiment is conducted with different *α* values, 0.9 and 0.5.  Figure 4 shows the adaptivity experimental result with respect to varying traffic intensity. Traffic intensity represents how many transmissions are generated during a *T*_TRA_. The result shows that LPL cycle dynamically adapts to the traffic variations. In particular, it is also shown that the larger the *α* value, the more adaptive the LPL cycle.

### 4.2. Response Delay

TRA-MAC is capable of minimizing response time by making the LPL cycle of a receiver and preamble length of a sender adaptive to the varying traffic. Therefore, to evaluate the effect of adaptive LC, we also observed average response delay of TRA devices and generic LPL devices, respectively, according to varying data traffic.

**Figure 5:**
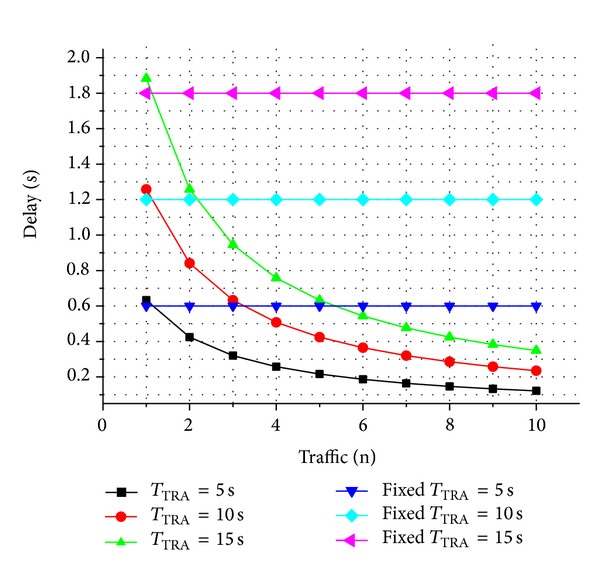
Response delay in uniform traffic.

Figure 5 shows delay distribution of TRA-MAC at different *T*_TRA_ (5, 10, and 15) and generic LPL at different fixed preamble length (5, 10, and 15), with respect to increasing data traffic from 1 to 10 at every interval. While generic LPL maintains almost the same delay without regard to the amount of traffics, TRA-MAC shows exponentially decreasing delay as the amount of data traffic increases. This result reveals that TRA-MAC ensures fast response time in sensor network environment having concurrent-intensive traffic pattern, by controlling dynamically preamble sensing cycle and preamble transmission length adaptively to the amount of data traffic.

### 4.3. Energy Consumption

We also measured energy consumption of TRA-MAC, compared to generic LPL with respect to varying the amount of traffics. To observe an effect with respect to different TRA duration, we experimented with different *T*_TRA_ (5–15).

**Figure 6:**
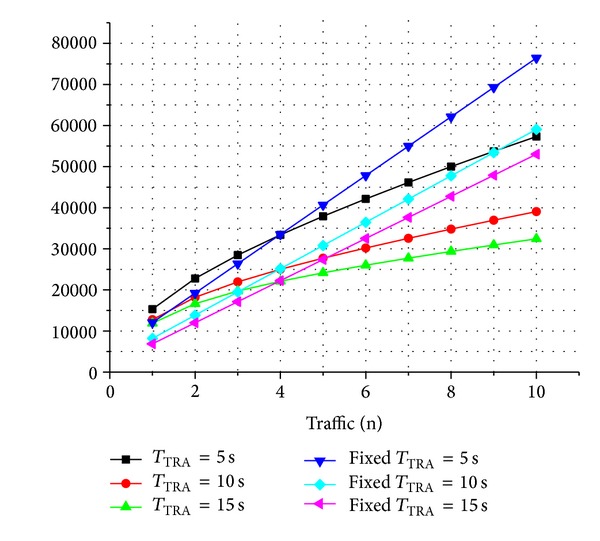
Energy consumption.

Figure 6 shows energy consumption of TRA-MAC and generic LPL device with respect to varying the amount of traffic, at different *T*_TRA_ (5, 10, and 15). As shown in Figure 6, energy consumption of generic LPL devices is linearly increasing as the amount of data traffic increases. On the other hand, energy of TRA-MAC devices is smoothly increasing compared to generic LPL. This is because TRA-MAC is capable of dynamically controlling LC and preamble length adaptively to transmission rates. In addition, the bounded LC length can reduce unnecessary energy wastage by minimizing continuous active period when transmission rates increase.

## 5. Conclusion

To minimize idle listening in WSN, so far there has been substantial research on low-power listening (LPL). Even though the existing LPL protocols have enabled ultra-low-power operation in WSN, they do not address trade-off between energy and delay, since they focused on energy efficiency itself. However, it is also important to meet various requirements, such as minimum delay and higher reliability, as well as energy efficiency. Therefore, in this paper, we introduced transmission rate based adaptive low-power listening MAC protocol (TRA-MAC), which is a kind of preamble-based LPL but is capable of adapting preamble sensing cycle dynamically based on transmission rate assessment. Through experiments with TRA prototype, it is demonstrated that TRA-MAC can dynamically control preamble sensing cycle and preamble transmission length adaptively to traffic variations. In particular, the TRA-MAC is designed to cope well with concurrent-intensive traffic environment in WSN by dealing with trade-off between delay and energy; the TRA-MAC will be more suitable for time critical sensor applications such as building infrastructure monitoring, security fields.

## Figures and Tables

**Table 1:** Types and brief features of LPL protocols.

LPL type	Protocol	Features
Preamble-based LPL	B-MAC [1]	(i) Performs check time, back-off window size, and power-down (ii) Utilizes CCA (clear channel assessment)
	Wise-MAC [2]	Enhanced LPL based on neighbors' schedule
	X-MAC [3]	Utilizes consecutive short preamble

Packet-based LPL	SpeckMAC [4]	(i) Two types: SpeckMAC-B and SpeckMAC-D (ii) Transmits consecutive data packet or wake-up packet
	BoX-MAC [5]	(i) Cross layer MAC protocol between PHY and MAC layer (ii) Two types: BoX-MAC-1 and BoX-MAC-2
	MX-MAC [6]	(i) MAC protocol using CSMA-MPS (ii) Compatible with X-MAC, SpeckMAC (iii) Transmits consecutive data packet

Receiver-initiated LPL	RI-MAC [7]	(i) Receiver transmits periodic beacon frame (ii) Sender transmits data after listening to receiver's beacon
	A-MAC [8]	(i) Utilizes hardware ACK (HACK) (ii) Based on neighbors' schedule

**Table:** (a) TRA at *T*(0) (TRA duration = 10 sec)

	*Prev_tcnt *	*Cur_tcnt *	LC
Device (1)	3	2	3.25
Device (2)	2	3	2.58
Device (3)	2	1	4.83

**Table:** (b) TRA at *T*(1) (TRA duration = 10 sec)

	*Prev_tcnt *	*Cur_tcnt *	LC
Device (1)	2	1	4.83
Device (2)	3	4	2.13 (LB)
Device (3)	1	1	5 (UB)

**Table:** (c) TRA at *T*(2) (TRA duration = 10 sec)

	*Prev_tcnt *	*Cur_tcnt *	LC
Device (1)	1	1	5 (UB)
Device (2)	4	1	4.7
Device (3)	1	1	5 (UB)

## Notations

LC_(*i*)_: Device *i*'s LPL cycle (variable)
*T*_PSD_: Preamble sensing duration (msec) (0.0156 msec (experiment))
LC_min⁡_: Unit LPL cycle (msec) (2∗PSD)
*L*_preamble_: Preamble transmission length (msec) (LC_(*i*)_ + 2∗PSD)
*α*: Weight coefficient (variable)
*Cu**r*_*tcnt*_(*i*)_: Device *i*'s current transmission count (variable).
*T*_DUD_: DMT update duration (variable)
*T*_TRA_: TRA duration (variable)
*Pr**ev*_*tcnt*_(*i*)_: Device *i*'s previous transmission count (variable)
MDT: Maximum delay tolerance (variable (application specific))
LB: Lower bound (LC_min⁡_)
UB: Upper bound (MDT)
*I*_*A*_: Current consumption in active state (0.0061944 mA (experiment))
*I*_*S*_: Current consumption in active state (0.0000083 mA (experiment))
V: Supply voltage (3.5 V).

## References

[1] Polastre J, Hill J, Culler D Versatile low power media access for wireless sensor networks.

[2] El-Hoiydi A, Decotignie J WiseMAC: an ultra low power MAC protocol for the downlink of infrastructure Wireless Sensor networks.

[3] Buettner M, Yee GV, Anderson E, Han R X-MAC: a short preamble MAC protocol for duty-cycled wireless sensor networks.

[4] Wong K-J, Arvind D SpeckMAC: low-power decentralized MAC protocol low data rate transmissions in Specknets.

[5] Moss D, Levis P (2008). BoX-MAC: exploiting physical and link layer boundaries in low-power networking.

[6] Merlin CJ, Heinzelman WB (2010). Schedule adaptation of low-power-listening protocols for wireless sensor networks. *IEEE Transactions on Mobile Computing*.

[7] Sun Y, Gurewitz O, Johnson DB RI-MAC: a receiver-initiated asynchronous duty cycle MAC protocol for dynamic traffic loads in wireless sensor networks.

[8] Dutta P, Dawson-Haggerty S (2012). Design and evaluation of a versatile and efficient receiver-initiated link layer for low-power wireless. *TOSN: ACM Transactions on Sensor Networks*.

[9] Hwang K-I, Jang I Ultra low power data aggregation for request oriented sensor
networks.

[10] Sinha A, Lobiyal DK (2013). Performance evaluation of data aggregation for cluster-based wireless sensor network. *Human-Centric Computing and Information Sciences*.

[11] Yoon M, Kim YK, Chang JW (2013). An energy-efficient routing protocol using message success rate in wireless sensor networks. *Journal of Convergence*.

